# Baseline Knee Osteoarthritis and Chronic Obstructive Pulmonary Disease as Predictors of Physical Activity Decline: A Five-Year Longitudinal Study in U.S. Adults Using the Disablement Process Framework

**DOI:** 10.3390/healthcare13151902

**Published:** 2025-08-05

**Authors:** Saad A. Alhammad, Vishal Vennu

**Affiliations:** Department of Rehabilitation Science, College of Applied Medical Sciences, King Saud University, P.O. Box 10219, Riyadh 11433, Saudi Arabia; shammad@ksu.edu.sa

**Keywords:** chronic conditions, physical activity, longitudinal study, PASE, rehabilitation, Osteoarthritis Initiative

## Abstract

**Background/Objective:** Understanding how chronic conditions such as knee osteoarthritis (OA) and chronic obstructive pulmonary disease (COPD) influence long-term physical activity (PA) is essential for developing condition-specific rehabilitation strategies. This study aimed to examine whether baseline diagnoses of knee OA and COPD are independently associated with the trajectories of PA decline over five years in U.S. adults, informed by the disablement process model. **Methods:** We analyzed data from 855 adults aged ≥45 years enrolled in the Osteoarthritis Initiative (OAI). The participants were categorized into three baseline groups, control (*n* = 122), knee OA (*n* = 646), and COPD (*n* = 87), based on self-reports and prior clinical assessments. PA was measured annually for five years using the Physical Activity Scale for the Elderly (PASE). General linear mixed models assessed changes in PA over time, adjusting for demographic, behavioral, and clinical covariates. **Results:** Compared to the controls, participants with knee OA had a significant decline in PA over time (β = −6.62; 95% CI: −15.4 to −2.19; *p* = 0.014). Those with COPD experienced an even greater decline compared to the knee OA group (β = −11.2; 95% CI: −21.7 to −0.67; *p* = 0.037). These associations persisted after adjusting for age, sex, body mass index, comorbidities, and smoking. **Conclusions:** Baseline knee OA and COPD were independently associated with long-term reductions in PA. These findings underscore the importance of early, tailored rehabilitation strategies, particularly pulmonary rehabilitation, in preserving functional independence among older adults with chronic conditions.

## 1. Introduction

Knee osteoarthritis (OA) and chronic obstructive pulmonary disease (COPD) are among the most prevalent chronic conditions that contribute to the long-term disability and healthcare burden globally and in the United States (U.S.). OA affects approximately 32.5 million U.S. adults [1], with knee OA ranking as the 11th highest contributor to global disability and 38th in disability-adjusted life years [2]. COPD affects over 16 million people, with U.S. prevalence estimated at 6.2%, and it disproportionately impacts older adults, smokers, and lower-income populations [3,4].

While both OA and COPD are independently associated with poor physical function and diminished quality of life, their impact on the long-term physical activity (PA) trajectories remains less clearly understood. Physical inactivity is a critical pathway through which chronic diseases exacerbate functional limitations, and it is a well-known modifiable risk factor for further disability, multimorbidity, and premature mortality [5,6]. From a theoretical perspective, the disablement process model [7] provides a useful lens for understanding how chronic conditions lead to functional limitations and reduced participation in life activities through intermediary processes, such as impairments and activity restrictions [8].

Knee OA contributes to activity limitation through joint pain, stiffness, reduced range of motion, and muscle weakness, leading many patients to avoid movement, thus perpetuating a cycle of deconditioning [9,10]. COPD, on the other hand, limits activity primarily through respiratory impairments, particularly dyspnea, which discourages sustained exertion and leads to progressive muscle loss and fatigue [11]. Both conditions represent prototypical models of physical disability—musculoskeletal and respiratory—and offer insight into how distinct mechanisms contribute to declining PA in aging populations.

Although previous studies have examined the cross-sectional relationships between OA or COPD and PA [12,13], few have looked at how activity levels change over multiple years. One prospective study by Yu et al. [14] followed patients with COPD for five years and found significant declines in PA. However, that study was limited to European participants, did not account for important sociodemographic or lifestyle factors like body mass index (BMI) or smoking, and did not include comparisons with musculoskeletal conditions such as OA.

To our knowledge, no study has simultaneously examined and compared the independent long-term effects of baseline knee OA and COPD on PA decline in a U.S. population. This represents an important gap, especially considering that both conditions often co-occur with aging and share common risk factors such as obesity and socioeconomic disadvantage [15,16].

Therefore, grounded in the disablement process model and chronic illness behavior theory, this study examines whether initial diagnoses of knee OA and COPD are independently linked to a five-year decline in PA among adults aged 45 years and older in the U.S. Using data from the Osteoarthritis Initiative (OAI), a large, multicenter longitudinal cohort, we aim to (1) measure the trajectory of PA decline in each condition; (2) compare these trajectories between OA and COPD groups; and (3) assess whether these associations remain after adjusting for clinical and sociodemographic confounders. By identifying condition-specific patterns of PA decline, we hope to inform tailored, early rehabilitation approaches to maintain mobility and independence among older adults.

## 2. Materials and Methods

### 2.1. Study Design and Setting

This was a longitudinal cohort study based on data from the OAI, an ongoing, publicly available multicenter study aimed at identifying risk factors for the progression of knee OA. The OAI enrolled 4796 adults aged 45 to 79 years between February 2004 and May 2006 across four U.S. clinical sites: Baltimore, MD; Columbus, OH; Pittsburgh, PA; and Pawtucket, RI. Data from the participants were collected annually and are available through the National Institutes of Health (NIH) public repository. This study analyzed data from the baseline through to the five-year follow-up (2004–2009).

The location of enrollment sites may influence the socioeconomic characteristics of the study sample, potentially affecting generalizability. Although the OAI recruited participants from diverse racial and ethnic backgrounds, the majority resided in urban or suburban regions with variable access to healthcare and recreational resources. These regional differences could influence health behaviors, including attitudes toward PA and adherence to clinical advice.

### 2.2. Study Participants

**Figure 1:**
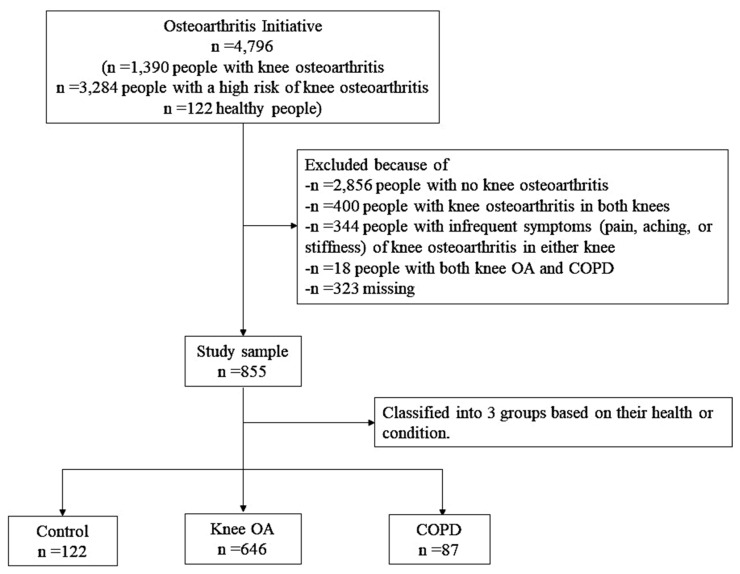
Flowchart of the study sample.

The flow of the study sample is presented in Figure 1. From the total OAI sample, we included 855 adults who had complete data on the key variables of interest and met the inclusion criteria. The participants were categorized into three baseline groups: the knee OA cohort (*n* = 646), COPD cohort (*n* = 87), and control cohort (*n* = 122). The knee OA cohort consists of participants who reported a physician diagnosis of OA or degenerative arthritis of the knee. The COPD cohort consists of participants who reported being diagnosed with emphysema, chronic bronchitis, or COPD in the past year. The control cohort consists of participants who reported neither of the above diagnoses.

We excluded participants who (a) had bilateral knee OA (*n* = 400); (b) reported both COPD and knee OA (*n* = 18); (c) had no knee OA (*n* = 2856); (d) had infrequent symptoms (such as pain, aching, or stiffness) of OA in either knee (*n* = 344); or (e) had missing values for the key covariates or outcome variables (*n* = 323). Participants with coexisting OA and COPD were excluded due to the small sample size, which precluded statistically meaningful subgroup analysis and could compromise model stability. Nevertheless, this exclusion is acknowledged as a limitation in the Discussion, given the high prevalence of multimorbidity in aging populations. No imputation was applied for missing data; only complete cases were included.

### 2.3. Diagnosis of Knee OA and COPD

Knee OA and COPD were classified using self-reported responses to standardized questions administered at the baseline. Knee OA was assessed using “Has a doctor ever told you that you have osteoarthritis or degenerative arthritis in your knee(s)?”. COPD was defined using “In the past twelve months, have you been diagnosed with emphysema, chronic bronchitis, or COPD?”. These diagnostic questions were adapted from previously validated epidemiologic surveys [17], and similar approaches have been used in large cohort studies with older adults [18]. However, the use of self-report introduces the possibility of misclassification bias, particularly given that clinical confirmation was not required for group assignment.

Although the OAI provides radiographically confirmed data for knee OA, comparable clinically verified data for COPD are not uniformly available. Therefore, self-reported diagnoses were used for both conditions to ensure consistency in terms of classification and avoid differential misclassification. These self-reports are based on validated survey instruments and have demonstrated reasonable accuracy in population-based studies [19,20].

### 2.4. Outcome Measure: Physical Activity

The researchers used the Physical Activity Scale for the Elderly (PASE) [21] to assess PA annually for five years. The PASE is a validated 12-item questionnaire that quantifies self-reported activity across three domains: leisure, household, and occupational. Higher PASE scores indicate greater PA, with a typical range from 0 to >400. The measure has been validated in older adult populations, including those with knee OA and COPD [22]. Data collection was standardized across all OAI sites using trained assessors.

### 2.5. Covariates

Covariates were selected based on the disability process model and the prior literature linking them to PA, chronic disease outcomes, or both [8,23,24]. These included demographics such as age (continuous), sex (male/female), race (white vs. non-white), marital status (married vs. not married), and education (≥high school vs. <high school). Socioeconomic status included employment (employed vs. unemployed) and annual household income (<USD 50,000 vs. ≥USD 50,000). Health behaviors included smoking status (current/former smoker vs. nonsmoker). Clinical variables included BMI (categorized as normal [18.5–24.9], overweight [25–29.9], or obese [≥30]) and the Charlson Comorbidity Index (0, 1, or ≥2 comorbidities). These variables were included as potential confounders because they influence both the presence/severity of OA and COPD and the likelihood of maintaining PA over time.

### 2.6. Statistical Analysis

Descriptive statistics (means, standard deviations, and proportions) were calculated for participant characteristics. Between-group differences were tested using Student’s independent *t*-test or an analysis of variance (ANOVA) for continuous variables and chi-square tests for categorical variables. To assess the changes in the model in terms of PA over time, we used general linear mixed models with an unstructured covariance matrix to account for intra-individual correlation across time points and unequal variances. This modeling approach is appropriate for handling unbalanced longitudinal data and assumes that missing data are missing at random. We did not impute the missing PASE scores.

Two models were constructed. Model 1 included time, group (knee OA, COPD, control), age, sex, race, marital status, education, employment, and income. Model 2 included all of the variables in Model 1, plus smoking status, BMI category, and the Charlson Comorbidity Index. Estimates were reported as beta coefficients with 95% confidence intervals (CIs). Model assumptions (normality of residuals, linearity, homoscedasticity) were assessed using residual plots and were found to be satisfactory.

To explore whether the association between condition and PA varied by participant characteristics, we conducted stratified analyses by age group (<65 vs. ≥65 years), sex, and BMI category. All analyses were conducted using SAS software version 9.4 (SAS Institute Inc., Cary, NC, USA). Statistical significance was set at *p* < 0.05.

## 3. Results

### 3.1. Summary of Participant Groups

Descriptive statistics for age, sex, race, education, income, BMI, and comorbidity burden are presented in Table 1. The average age of the participants was 60.4 years. Of the cohort, 75.5% were diagnosed with knee OA, 10.2% with COPD, and 14.3% were in the control group. Females represented the majority in both the knee OA and COPD groups, accounting for 51.9% and 63.2% of the respective cohorts. Caucasians had higher knee OA and COPD rates than other races (75.4 and 78.2%, respectively). Most people with COPD (68.5%) or knee OA (82.7%) had only primary education. People with COPD were more likely to be unemployed (62.1%), current or former smokers (68.6%), have a yearly income of less than USD 50,000 (55.2%), and have one or more comorbid conditions (69%). Most people with knee OA or COPD were overweight (75.9%) or obese (86.7%). On average, patients with COPD engaged in less PA (PASE = 137.8) (Table 1).

### 3.2. Physical Activity Trajectories over Time

The general linear mixed models revealed that participants with either knee OA or COPD experienced declines in PA over the five years, though the patterns and magnitude differed between the groups. In the unadjusted analysis, there was a visible downward trend in the PASE scores in both the knee OA and COPD groups, while the scores in the control group remained relatively stable. Compared to the control group, participants with knee OA had significantly lower PA over time (β = −9.44; 95% CI: −17.7 to −1.09; *p* = 0.027). Participants with COPD had significantly lower PA compared to those with knee OA (β = −16.9; 95% CI: −26.7 to −7.17; *p* = 0.007), indicating a more substantial decline.

**Figure 2:**
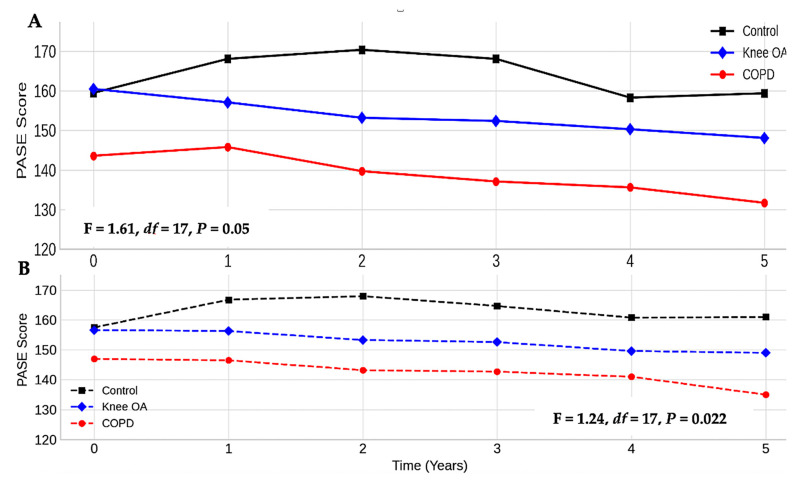
Average physical activity over five years. (**A**) Adjusted for age, sex, race, marital status, educational level, employment status, and annual income; (**B**) adjusted for age, sex, race, marital status, educational level, employment status, annual income, smoking status, comorbidity, and body mass index.

When further adjusted for smoking status, BMI, and comorbidities, these associations persisted: knee OA vs. control (β = −6.62; 95% CI: −15.4 to −2.19; *p* = 0.014) and COPD vs. knee OA (β = −11.2; 95% CI: −21.7 to −0.67; *p* = 0.037). There was no significant effect of time alone (β = −0.10; 95% CI: −0.52 to 0.60; *p* = 0.895), indicating that the declines in the PASE scores were driven more by the baseline condition group than by time itself (Table 2 presents the full model results. Figure 2A,B illustrate the adjusted PASE score trajectories).

### 3.3. Subgroup and Interaction Analyses

Among participants aged ≥65, the decline in PA was more pronounced for both the OA and COPD groups compared to those under 65. The effect of COPD on activity decline remained significant in this older subgroup (β = −13.7; 95% CI: −24.3 to −3.10; *p* = 0.012). Female participants with COPD experienced greater reductions in the PASE scores than males with COPD, although the interaction term was not statistically significant (*p* for interaction = 0.091). The negative association between COPD and PA was strongest among obese participants (β = −15.2; 95% CI: −25.9 to −4.46; *p* = 0.006). No statistically significant interaction terms were detected, but the effect sizes suggested clinically meaningful patterns by subgroup.

### 3.4. Model Diagnostics and Sensitivity Checks

Residual plots indicated the normal distribution of residuals and homoscedasticity. No multicollinearity was observed (variance inflation factors < 2). The exclusion of outliers did not materially alter the effect estimates. A sensitivity analysis reclassifying borderline BMI cases did not change the statistical significance of the COPD effect.

## 4. Discussion

This study investigated the longitudinal relationship between baseline diagnoses of knee OA and COPD and PA decline over five years among U.S. adults. Guided by the disablement process model, our findings reveal that both individuals with OA and COPD are independently associated with significant reductions in PA, even after adjusting for socioeconomic and health-related confounders. Importantly, participants with COPD experienced a more pronounced and persistent decline in PA compared to those with OA, underscoring the greater functional impact of pulmonary limitations on mobility trajectories in aging populations.

These findings are consistent with and extend prior evidence. In line with Yu et al.’s study [14], who demonstrated a 5-year PA decline in European COPD patients, our U.S.-based study confirms similar trends in a more diverse population and with adjustment for critical covariates such as comorbidities and obesity, factors not accounted for in Yu et al.’s study [14]. Moreover, by including a comparison with a musculoskeletal condition, such as OA, this research uniquely contributes to the literature by illustrating how different chronic conditions influence PA patterns through distinct pathophysiological and behavioral pathways.

From a conceptual perspective, the disablement process model provides a useful lens through which to understand these findings. In OA, impairments such as joint pain, stiffness, and reduced range of motion initiate a cascade of functional limitations that deter voluntary movement [25]. For COPD, respiratory impairments such as dyspnea and airway remodeling limit exercise tolerance and result in muscle deconditioning, further exacerbating activity avoidance [26]. This explains why, in our cohort, COPD patients—who were older, more obese, and more likely to have multiple comorbidities—demonstrated both lower baseline PASE scores and steeper declines.

The finding that PA declined more substantially in COPD than OA also reflects the cumulative impact of systemic deconditioning [27]. Whereas OA patients may retain some ability to engage in adapted or lower-impact activities, COPD-related breathlessness often limits any exertion, particularly in older, multimorbid, or obese individuals [28]. Stratified analyses revealed that this pattern was especially pronounced in older adults and those with obesity, two groups that often face overlapping barriers to PA, including fatigue, limited access to supportive environments, and functional impairments.

Our results have important clinical and policy implications. First, the early identification of PA decline should be a priority in routine care for patients with COPD or OA. In primary care settings, validated screening tools such as PASE can be integrated into annual wellness visits to track activity trends. Second, pulmonary rehabilitation programs must be initiated early, ideally upon diagnosis, rather than in late-stage disease [29]. Interventions should include aerobic conditioning, muscle strengthening, and behavioral support to improve adherence and overcome barriers such as fatigue or depression [30].

Third, there is an urgent need to link PA promotion strategies to active aging policies and chronic disease management guidelines [31]. For instance, multidisciplinary care models that incorporate physical therapists, occupational therapists, and health coaches could improve program delivery. A practical target of 5000 steps/day [32], as identified in prior COPD research, may be both feasible and beneficial, especially if embedded within personalized care plans. Finally, given the socioeconomic disparities observed in our sample, interventions must be tailored to reach lower-income or unemployed adults, for whom structural barriers may limit engagement in PA.

### 4.1. Study Limitations

This study has several limitations that warrant consideration: First, self-reported diagnoses. Both knee OA and COPD were identified via self-report, which introduces the potential for misclassification. Although the questions were adapted from validated surveys [19,20], the absence of clinical confirmation may reduce diagnostic accuracy, particularly among older adults with cognitive or recall issues [33]. Second, PA measurement. The PASE scores are also self-reported and thus susceptible to recall bias and social desirability effects. While validated and widely used in geriatric populations [34], they do not capture objective movement data such as step counts or accelerometer-derived intensity. Third, the exclusion of multimorbid participants. Individuals with both knee OA and COPD (*n* = 18) were excluded due to an insufficient sample size for subgroup analysis. However, this exclusion limits the generalizability of our findings, particularly in light of the growing prevalence of multimorbidity in older adults. Future studies should consider pooling data from multiple cohorts or employing advanced statistical approaches—such as latent class modeling or multimorbidity pattern recognition—to adequately assess the impact of overlapping chronic conditions, including the combined effects of OA and COPD on PA trajectories. Fourth, sample generalizability. Although the OAI recruited from four U.S. clinical sites and included participants of different races and education levels, most were from urban or suburban settings, potentially limiting applicability to rural or underserved populations. Fifth, residual confounding. Despite adjusting for key covariates (e.g., age, BMI, smoking, comorbidities), unmeasured factors, such as depression, access to recreational facilities, or neighborhood walkability, may have influenced the PA trajectories. Finally, missing data. While mixed models allow for some handling of missing data under the missing-at-random assumption, we did not perform multiple imputations. Thus, non-random missingness could still introduce bias. Despite these limitations, this study’s strengths—including a large sample, five-year follow-up, validated PA measure, and theoretically informed analysis—support the robustness of our findings.

### 4.2. Public Health Relevance

The observed decline in PA among older adults with knee OA and especially those with COPD has important public health implications, particularly in the context of a rapidly aging global population. Both conditions contribute significantly to disability-adjusted life years and healthcare utilization, and our findings highlight their role in accelerating functional decline. Promoting and preserving PA in these populations is not only vital for maintaining independence and quality of life but also for reducing the long-term burden on healthcare systems. These results support the integration of condition-specific PA strategies into national chronic disease prevention and healthy aging policies. Scalable interventions—such as community-based walking programs, pulmonary rehabilitation in primary care, and digital coaching platforms—could be prioritized for implementation in public health systems. Furthermore, targeted efforts to reach socially and economically disadvantaged older adults are essential to reduce disparities and promote equitable access to activity-enhancing resources. In sum, our findings emphasize the need for preventive, rehabilitative, and policy-level actions to mitigate PA decline in older adults with chronic diseases and support healthier aging trajectories.

## 5. Conclusions

This longitudinal study demonstrates that baseline diagnoses of knee OA and COPD are independently associated with significant declines in PA over five years among U.S. adults. Importantly, COPD was associated with a more pronounced and sustained reduction in activity compared to knee OA, likely reflecting the compounding effects of respiratory impairment, systemic deconditioning, and comorbid burden. Grounded in the disability process model, these findings highlight the critical need for early, condition-specific intervention strategies. In particular, integrating pulmonary rehabilitation and tailored PA programs into routine care—especially in primary care settings—may help preserve mobility and prevent functional decline among older adults with chronic conditions. Health systems should prioritize systematic screening for activity decline, promote accessible exercise interventions, and support active aging policies that reduce disparities in physical functioning. Future research should address the needs of multimorbid populations, incorporate objective PA measurements, and examine the role played by additional modifiable risk factors such as nutrition, mental health, and environmental supports. Intervening early in the disablement trajectory holds promise for maintaining independence, reducing healthcare costs, and improving quality of life in aging populations.

## Figures and Tables

**Table 1:** Characteristics of the study participants.

Characteristics	Groups			*p*
	Control *n* = 122 (14.3%)	Knee OA *n* = 646 (75.5%)	COPD *n* = 87 (10.2%)	
Age in a year, mean (SD)	55.0 (7.6)	61.1 (8.4)	65.1 (8.7)	<0.001 ***
Age group, *n* (%)				<0.001 *
<65 years	40 (46)	407 (63)	105 (86.1)	
≥65 years	47 (54)	239 (37)	17 (13.9)	
Sex, *n* (%)				0.032 *
Male	47 (38.5)	311 (48.1)	32 (36.8)	
Female	75 (61.5)	335 (51.9)	55 (63.2)	
Race, *n* (%)				0.001 *
White or Caucasians	113 (92.6)	487 (75.4)	68 (78.2)	
Black or African American	9 (7.4)	159 (24.6)	19 (21.8)	
Marital status, *n* (%)				0.002 *
Married	100 (82)	426 (65.9)	56 (64.4)	
Unmarried/divorced/widowed	22 (18)	220 (34.1)	31 (35.6)	
Educational status, *n* (%)				<0.001 *
Primary school or less	5 (4.1)	534 (82.7)	60 (68.9)	
High school or more	117 (95.9)	112 (17.3)	27 (31.1)	
Employment status, *n* (%)				<0.001 *
Employed	106 (86.9)	379 (58.7)	33 (37.9)	
Unemployed	16 (13.1)	267 (41.3)	54 (62.1)	
Annual income, *n* (%)				<0.001 *
<USD 50,000	18 (14.7)	224 (34.7)	48 (55.2)	
≥USD 50,000	104 (85.3)	422 (65.3)	39 (44.8)	
Smoking status, *n* (%)				<0.001 **
Nonsmoker	81 (66.9)	309 (48.7)	27 (31.4)	
Current smoker	7 (5.8)	279 (44)	13 (15.1)	
Former smoker	33 (27.3)	46 (7.3)	46 (53.5)	
Comorbidity, *n* (%)				<0.001 **
0	112 (92.6)	465 (73.8)	27 (31)	
1	5 (4.1)	110 (17.5)	40 (46)	
≥2	4 (3.3)	55 (8.7)	20 (23)	
BMI (kg/m^2^), *n* (%)				<0.001 **
Normal weight	70 (58.3)	155 (24.1)	12 (13.8)	
Overweight	44 (36.7)	264 (35)	32 (36.8)	
Obese	6 (5)	226 (40.9)	43 (49.4)	
PASE, mean (SD)	178.6 (75.9)	158.4 (80.1)	130.7 (68.7)	<0.001 ***

Abbreviations: OA, osteoarthritis; COPD, chronic obstructive pulmonary disease; OA, osteoarthritis; BMI, body mass index; PASE, Physical Activity Scale for the Elderly. * Chi-square test. ** Analysis of variance test. *** Student’s independent *t*-test.

**Table 2:** Relationship between knee osteoarthritis, chronic obstructive pulmonary disease, and long-term physical activity in U.S. adults.

Variable	Model 1 ^a^			Model 2 ^b^		
	*β*	95% CI	*p*	*β*	95% CI	*p*
Intercept	211.4	203.1, 219.7	<0.001	214.6	204.8, 224.4	<0.001
Time	−0.11	−1.45, 0.67	0.708	−0.10	−0.52, 0.60	0.895
Knee OA versus control	−9.44	−17.7, −1.09	0.027	−6.62	−15.4, −2.19	0.014
COPD versus knee OA	−16.9	−26.7, −7.17	0.007	−11.2	−21.7, −0.67	0.037

Abbreviations: OA, osteoarthritis; CI, confidence interval; COPD, chronic obstructive pulmonary disease. ^a^ Model 1 adjusted for time, cohorts, age, sex, race, education, marital status, employment, and annual income. ^b^ Model 2 adjusted for Model 1 plus smoking, BMI, and comorbidities.

## Data Availability

The data associated with this paper are available in the Osteoarthritis Initiative via https://data-archive.nimh.nih.gov/oai/ (accessed on 2 August 2025).

## Abbreviations

The following abbreviations are used in this manuscript:

COPD	Chronic obstructive pulmonary disease
OA	Osteoarthritis
PASE	Physical Activity Scale for the Elderly
BMI	Body mass index
PA	Physical activity
